# *Mycoplasma ovipneumoniae* - A Primary Cause of Severe Pneumonia Epizootics in the Norwegian Muskox (*Ovibos moschatus*) Population

**DOI:** 10.1371/journal.pone.0106116

**Published:** 2014-09-08

**Authors:** Kjell Handeland, Torstein Tengs, Branko Kokotovic, Turid Vikøren, Roger D. Ayling, Bjarne Bergsjø, Ólöf G. Sigurðardóttir, Tord Bretten

**Affiliations:** 1 Norwegian Veterinary Institute, Oslo, Norway; 2 National Veterinary Institute, Copenhagen, Denmark; 3 Animal Health and Veterinary Laboratories Agency (Weybridge), Addlestone, Surrey, United Kingdom; 4 Institute for Experimental Pathology, University of Iceland, Keldur, Iceland; 5 Norwegian Nature Inspectorate, Oppdal, Norway; Virginia Tech University, United States of America

## Abstract

The Norwegian muskox (*Ovibos moschatus*) population lives on the high mountain plateau of Dovre and originates from animals introduced from Greenland. In the late summers of 2006 and 2012, severe outbreaks of pneumonia with mortality rates of 25-30% occurred. During the 2012 epidemic high quality samples from culled sick animals were obtained for microbiological and pathological examinations. High throughput sequencing (pyrosequencing) of pneumonic lung tissue revealed high concentrations of *Mycoplasma ovipneumoniae* in all six animals examined by this method and *Pasteurella multocida* subsp. *multocida* in four animals, whereas no virus sequences could be identified. *Mycoplasma ovipneumoniae* and *P. multocida multocida* were also isolated by culture. Using real time PCR on lung swabs, *M. ovipneumoniae* was detected in all of the 19 pneumonic lungs examined. Gross pathological examination revealed heavy consolidations primarily in the cranial parts of the lungs and it also identified one case of otitis media. Histologically, lung lesions were characterized as acute to subacute mixed exudative and moderately proliferative bronchoalveolar pneumonia. Immunohistochemical (IHC) examination revealed high load of *M. ovipneumoniae* antigens within lung lesions, with particularly intensive staining in the neutrophils. Similar IHC finding were observed in archived lung tissue blocks from animals examined during the 2006 epidemic. An *M. ovipneumoniae* specific ELISA was applied on bio-banked muskox sera from stray muskoxen killed in the period 2004–2013 and sick muskoxen culled, as well as sera from wild reindeer (*Rangifer tarandus tarandus*) on Dovre and muskoxen from Greenland. Serology and mycoplasma culturing was also carried out on sheep that had been on pasture in the muskox area during the outbreak in 2012. Our findings indicated separate introductions of *M. ovipneumoniae* infection in 2006 and 2012 from infected co-grazing sheep. Salt licks shared by the two species were a possible route of transmitting infection.

## Introduction

The muskox (*Ovibos moschatus*) is a bovid species belonging to the subfamily Caprinae, thus being more closely related to sheep and goats than to oxen. Until the end of the last glacial period, this species was present in many parts of the Holarctic, including Scandinavia. Then, it was brought to near extinction over large areas and its present native distribution is restricted to northern Canada and northeastern Greenland. Several successful introductions or re-introductions have been carried out in Alaska, western Greenland, Norway and Siberia [1].

**Figure 1:**
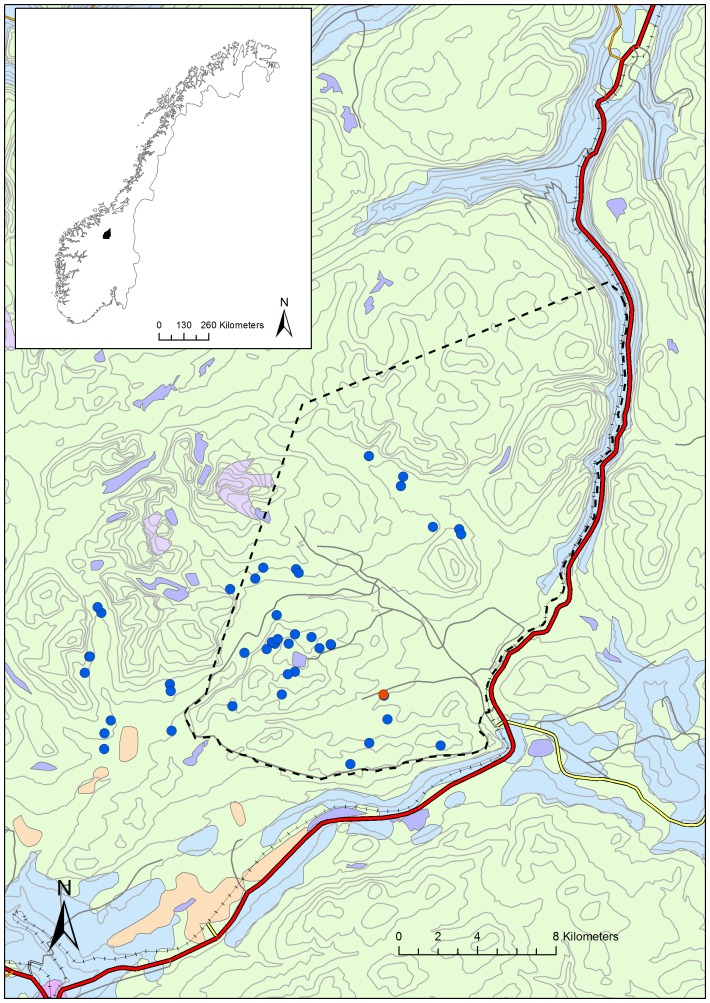
Norwegian muskox area and finding spots of sick animals. Map showing the geographic location (Insert: black dot) of the high mountain plateau of Dovre in South Norway (centre: 62°20′N, 09°30′E) and the home range (dotted line) of the Norwegian muskox population living on Dovre. The spots of detection of dead/sick muskoxen found during the 2012 pneumonia epidemic are indicated; the index case by a red symbol and the remainder by blue symbols.

The Norwegian muskox population originates from 21 muskox calves introduced from northeast Greenland between 1947 and 1953 and they live on the high mountain plateau of Dovre in South Norway (Fig. 1) [2]. During the first 40 years the population grew slowly, whereas there has been rapid growth from the late 1990s. In 1998, for the first time the summer population exceeded 100 animals. In 2006 there were more than 200 animals and in 2012 the population had increased to approximately 350. During the summer, domestic sheep and cattle are present in the southernmost part of the muskox area where the three species share salt licks set out for livestock. The area is also populated by one of Norway's wild reindeer (*Rangifer tarandus tarandus*) populations.

Three major disease outbreaks have been recognized within the Norwegian muskox population: An outbreak of contagious ecthyma in muskox calves in July-October 2004 [3], and two outbreaks of highly fatal pneumonia in the late summers of 2006 and 2012. A few pneumonia cases were also diagnosed in 2013. The 2006 epidemic affected all age groups when approximately 25% of the animals died [4] and the outbreak was thought to be caused by *Pasteurella/Mannheimia*-bacteria (pasteurellosis). However, samples obtained for microbiological examination in general were of poor quality and examinations for other possible underlying primary agents like respiratory viruses and mycoplasmas were not carried out.

During the 2012 pneumonia epidemic and from the occasional cases occurring in 2013, efforts were made to gather high quality microbiological samples from sick animals culled in the field. These samples were examined using conventional bacteriological methods and modern molecular-biological methods, including high throughput sequencing (pyrosequencing). The study also included retrospective examinations of archived lung tissues from the 2006 epidemic; and sera from muskoxen killed in the period 2004–2013; as well as mycoplasma examinations of sheep and reindeer from Dovre, and muskoxen from Greenland.

## Materials and Methods

### Surveillance and disease histories

The area defined as the muskox home range on Dovre consists of 340 km^2^ of alpine tundra which is bordered by the railroad and highway connecting southern and central parts of South Norway (Fig. 1). The population is monitored by the Norwegian Nature Inspectorate (NNI) and is counted at least once a year. The population is not harvested, however, animals that stray from their range are culled due to human security reasons and another important mortality factor is railroad accidents. From these animals, blood samples have been routinely collected since 2004 and the sera stored in biobank (Norwegian Veterinary Institute) at −40°C.

The index case of the 2012 outbreak was found dead on August 11. During the remainder of August and in September another 39 carcasses were detected and six sick animals were culled due to animal welfare reasons after approval from the Norwegian Environment Agency. All culling was carried out by trained NNI personal authorized to euthanize muskoxen. The animals were euthanized by a shot to the chest (normal hunting way) and sampled for examination/biobanking at the Norwegian Veterinary Institute. This sampling is performed routinely as part of the Health Surveillance Program for Muskox run by the Norwegian Veterinary Institute and funded by the Norwegian Environment Agency.

The majority of animals were found in the southern part of the muskox range (Fig. 1). Reindeer hunters and military personal, being active in the area were central in detection of cases that were subsequently recorded by the NNI [5]. Of the 46 animals recorded 36 (78%) were three-years old or less and the remainder were young adults. The surveillance and registration work ceased towards the end of September due to snowfall and the end of the reindeer hunting season. However, based upon the counts in July 2012 and March 2013, it was calculated that 30–35% of the animals had died during this epidemic. In August and September 2013, three sick (pneumonic) calves and one seemingly healthy stray yearling with pneumonic lungs were culled by the NNI.

**Figure 2:**
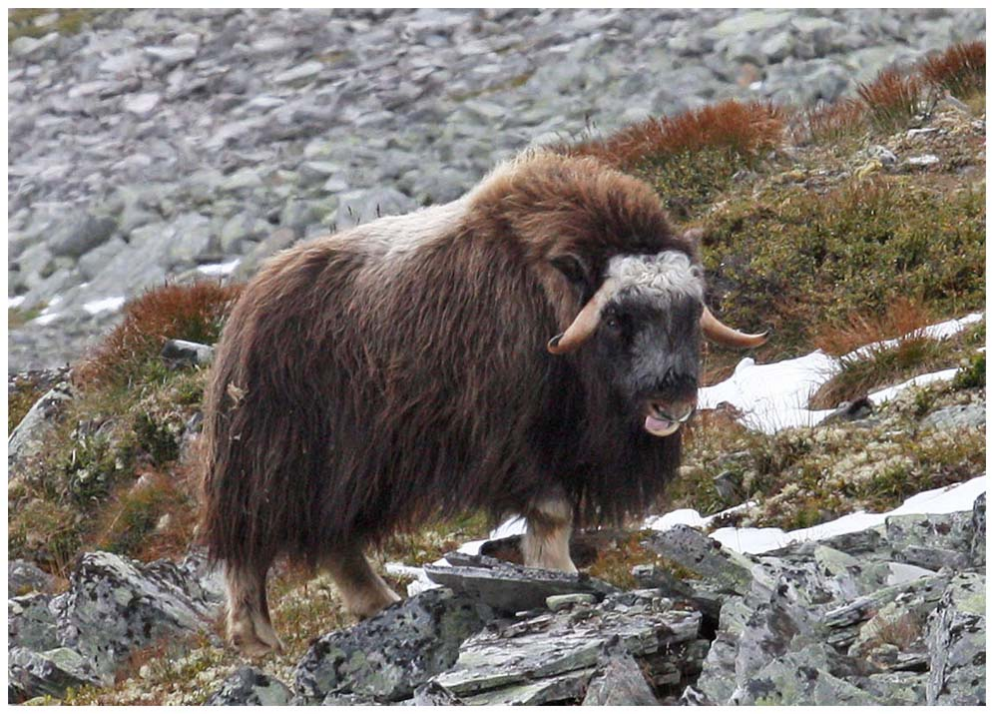
Sick pneumonic muskox. A young sick (pneumonic) muskox. Note the protruding tongue.

Sick muskoxen showed signs of lethargy, depression, reduced mobility and prolonged periods of lying down. Coughing was observed but was not a prominent finding. The respiratory rate was difficult to evaluate due to the thick hair coat. However, a regular pumping out of the tongue, presumably an attempt to facilitate respiration (inhalation), was commonly observed in sick animals (Fig. 2) and some of them had diarrhea. One animal showed a tendency of sideways drifting while moving whereas another had pus on one ear pinna.

### Pathology

A summary of the animals/materials examined by pathology is given in Table 1. Gross examination included the carcasses of four animals found dead and one sick animal culled and autopsied by veterinary pathologist in the field in 2012, as well as the lungs or lung tissue samples from eight sick animals culled and sampled by the NNI in 2012 and 2013. From one of the animals, its head was also submitted due to the presence of pus on one ear. Histology, and immunohistochemistry (IHC) against *Mycoplasma ovipneumoniae* were carried out on multiple lung specimens from all of the culled animals. From the animal culled and professionally autopsied in the field, these examinations also included pulmonary and six mesenteric (jejunal, ileocaecal) lymph nodes. IHC examination was also carried out on archived lung tissue blocks from seven pneumonic animals examined during the 2006 epidemic.

**Table 1:** Pathological examinations.

Category	Postmortem				Histology and IHC	
	Carcass	Lungs	Lung samples	Head	Lungs	Lymph nodes*
Dead 2012	4					
Culled 2012	1**		5		6	1
Culled 2013		3		1	3	

Materials examined by postmortem, histology and immunohistochemistry (IHC) against *Mycoplasma ovipneumoniae* from 13 pneumonic muskoxen found dead or culled in August/September 2012 and 2013.

*Pulmonary and mesenteric (ileocaecal, jejunal) lymph nodes.

**Culled and autopsied in the field September 5, 2012.

Tissues for histology were fixed in 10% neutral buffered formalin, embedded in paraffin, sectioned at 3–4 µm and stained with haematoxylin and eosin (HE). Additional staining methods applied included van Gieson for differential connective tissue staining, and Martius Scarlet Blue for demonstration of fibrin. IHC against *M. ovipneumoniae* was carried out, using the same primary polyclonal antibodies and methods as described earlier [6] with a few modifications: after antigen retrieval with 0.1% pronase, sections were incubated with normal goat serum and the primary antibodies were used at a dilution 1/500. In control sections, the *M. ovipneumoniae* antibodies were replaced with purified IgG (AbD Serotec) from non-immunized rabbits.

### Microbiology

Equipment and an instruction for microbiological field sampling were distributed to the NMI in late August 2012. Sampling included the swabbing and the extraction of a small tissue sample from the cut surface of one apical (consolidated) lung lobe. Two swabs were conserved in an in-house produced virus transport medium which contained penicillin, streptomycin and amphotericin B (BioNordica Bergman) and these swabs were used for mycoplasma real-time PCR (described later) and culturing. A third swab for conventional bacteriology was placed in bacteria transport medium (VWR, COPAN, Italy). The tissue sample was conserved in RNAlater (QIAGEN) and was used for pyrosequencing.

This sampling procedure was used on a total of 33 animals: Sick muskoxen culled in 2012 and 2013, moderately decomposed carcasses found in 2012, and stray animals culled in the period September 2012-October 2013. For each of these categories of animals, a selected number of microbiological examinations (pyrosequencing, conventional bacteriology, mycoplasma culturing, and *M. ovipneumoniae* real time PCR) were carried out (Table 2).

**Table 2:** Microbiological examinations.

Category	Pyrosequencing	Conventional bacteriology	*Mycoplasma* culturing	Real time PCR M.ovipneumoniae
Sick 2012	6	6	6/4*	6/6*
Sick 2013		3	3/1	3/3
Dead 2012				10/10
Stray animals				14/2

Microbiology, by method and animal category, as carried out on sick muskoxen culled in September 2012 and August/September 2013, muskoxen found dead in September 2012, and stray animals killed in the period September 2012-October 2013.

*Number of animals found positive for *Mycoplasma ovipneumoniae*.

For pyrosequencing, total RNA was extracted using the RNeasy kit (QIAGEN). The RNA was DNase treated using TURBO DNA-free (Applied Biosystems/Ambion, Austin, TX, USA), pooled and reverse transcribed/amplified using the QuantiTect kit (QIAGEN). Sequencing was done by the Norwegian Sequencing Centre using the Roche 454 GS-FLX system and Titanium chemistry (454 Life Sciences, a Roche company, Branford, CT, USA). Sequence reads were assembled using the MIRA sequence assembler (http://sourceforge.net/projects/mira-assembler/). Sequence contigs were computationally subtracted [7] using the sheep (*Ovis aries*) genome (GenBank accession: PRJNA179263) and all mitochondrial sequences from the Bovidae family in Genbank using the megablast algorithm [8]. All viral sequences in GenBank were also downloaded as well as all complete bacterial genomes. For sequence comparison against the viral database, translated protein searches were done using the program tblastx [9]. Bacterial searches were done using nucleotide-nucleotide BLAST and phylogenetic analyses were done using the program SeaView (version 4).

The *M. ovipneumoniae* real-time PCR was designed using the software Primer Express (version 2.0.0; Applied Biosystems, Life Technologies Corporation, Carlsbad, CA, USA) (forward primer Mo16S_35F: TGGGTGAGTAACACGTACCTAACC, reverse primer Mo16S_96R: AGCCGCTGTTTCCAATGG, FAM-labeled MGB probe Mo16S_60T: ACCTTTTGGACCGGGATA). Nucleic acids for PCR were extracted using the NucliSENS easyMAG DNA/RNA extraction system (bioMérieux, Inc, Durham, NC, USA) and PCR was performed using the HotStarTaq Master Mix kit (QIAGEN) with final concentration of primers and MGB probe 0.4 uM and the following PCR cycle: 95°C (15 min; polymerase activation) and 94°C (15 s)/55°C (30 s)/72°C (15 s) for 45 cycles.

Swabs for conventional bacteriology were plated out on one lactose-sucrose bromthymol blue agar and two bovine blood agar plates that were incubated for one day at 37°C aerobically, in 5% CO_2_ and anaerobically, respectively. The isolates were identified using standard bacteriological methods.

Swabs for *Mycoplasma* culturing were seeded into Friis' medium [10] and incubated at 37°C with daily control for color change. For identification, liquid cultures were plated onto solid medium and identified by disk growth inhibition (DGI) test using rabbit hyperimmune sera raised against *M. ovipneumoniae* Y-98.

### Serology

*Mycoplasma ovipneumoniae* serology was carried out by means of an indirect ELISA, using an optimum dilution of a whole cell antigen and a protein G peroxidase conjugate (Thermo Scientific, UK). Briefly, microtitre plates were coated for 2 hours at 37°C using a carbonate/bicarbonate buffer, with a 0.01M PBS washed isolate of *M. ovipneumoniae*, which had previously been selected following testing of sera from sheep flocks infected with different molecular types of *M. ovipneumoniae* as determined by pulsed field gel electrophoresis and random amplified polymorphic DNA [11]. The plates were washed with 0.01M PBS pH7.2, sera tested in duplicate was then added, diluted 1/100 with 0.01M PBS, with 0.25% tween 20 and 1% milk powder. The plate was incubated at 37°C for 30 minutes, washed and then incubated as before at 37°C for 30 minutes with the optimum dilution of protein G conjugate, washed and then chromogen substrate was added. When sufficient colour had developed in the positive control, the reaction was stopped using 1M Citric acid and the microtitre plate was read at OD_450_. The results were calculated to give the positive control an OD_450_ of 1.0 and OD_450_ values >0.35, used as cut off in sheep, were regarded as positive.

The ELISA examination included sera from 114 muskoxen stored in the HOP biobank during the period July 2004 to October 2013 (Table 3). The number of samples varied greatly between years with the majority originating from stray or railway-killed adult animals. In 2004, however, the number of calves sampled was high because of the culling of calves affected by contagious ecthyma [3]. Unfortunately, no serum samples were available from sick animals culled during the 2006 epidemic.

**Table 3:** Serological examination for *Mycoplasma ovipneumoniae*.

Category	July 2004-June 2006	July 2006-June 2012	July 2012- Oct 2013
Ecthyma-sick calves*	4/12		
Pneumonic animals**			10/10
Stray animals	1***/24	11/57	9/11

Number of muskoxen, by category and period, as found positive/examined for antibodies against *M. ovipneumoniae* by ELISA, 2004–2013.

*Culled in the period July-October 2004.

**Culled in August/September 2012 and 2013.

***Shot in July 2004.

Serology also included 96 muskoxen from Kangerlussuaq, West Greenland sampled during ordinary hunting in February 2014. This population originates from 27 animals introduced from northeast Greenland in the 1960s and constitute Greenland's largest and only ordinary hunted muskox population, with between 10,000–25,000 animals [1], [12]. The Greenland reindeer (*Rangifer tarandus groenlandicus*) is the only other ruminant present in the Kangerlussuaq area.

### Sheep and reindeer

Four sheep flocks that had been on pasture in the southern part of the muskox area during the summer of 2012 were sampled at the end of the housing season, in May 2013. The sampling included blood and two deep nasal swabs (UTM-kit, COPAN, Italy) taken from each nostril of 10 one-year-old sheep per flock. The same sampling procedure was carried out on 35 wild reindeer from Dovre shot during hunting in August-September 2013. Sheep samples were examined by *M. ovipneumoniae* ELISA and mycoplasma culturing, whereas the reindeer samples were only examined using serology.

## Results

### Pathology

**Figure 3:**
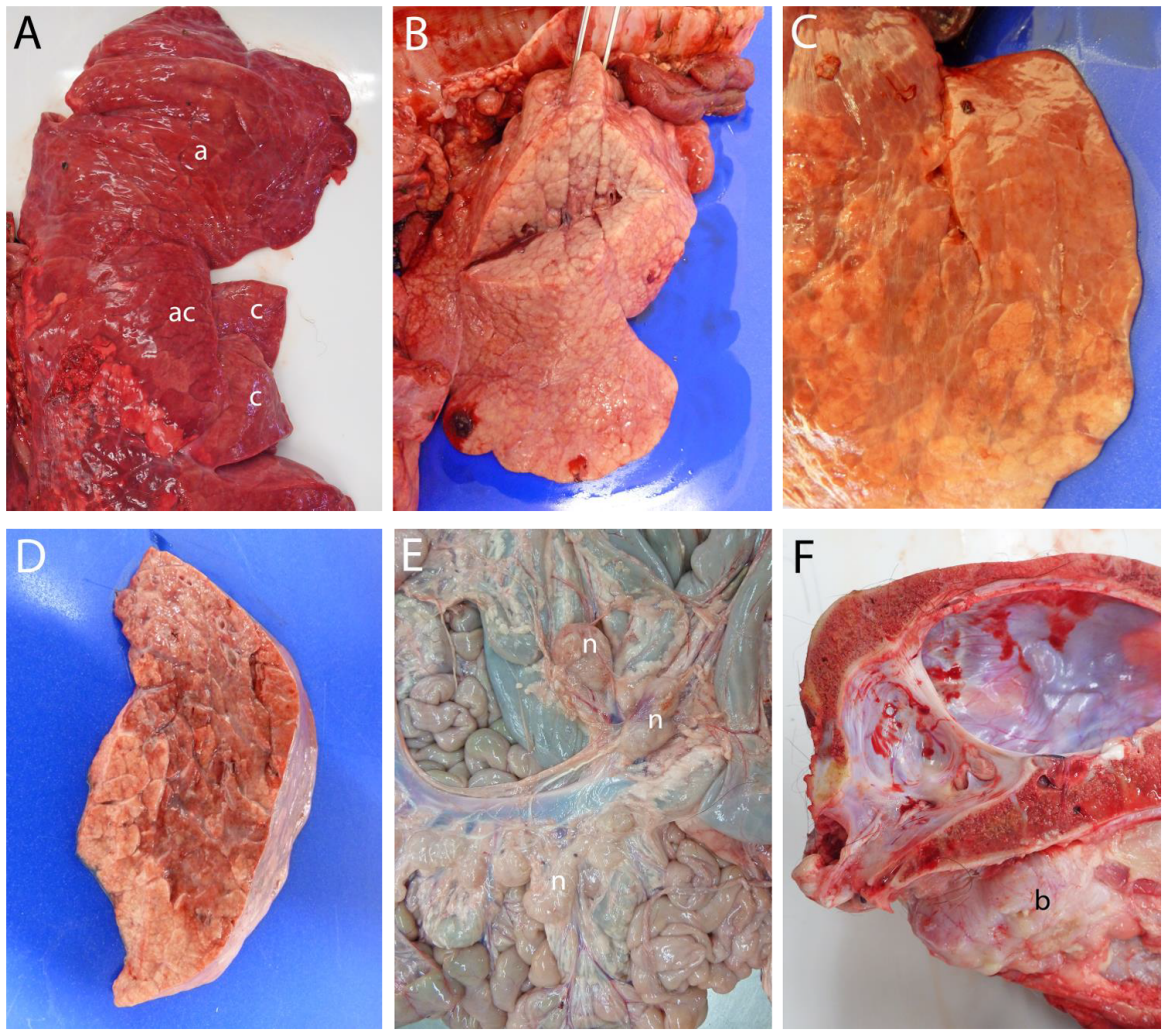
Gross lesions in sick muskoxen. (A) Right lung showing confluent red-grey consolidations (lobar pneumonia) in the apical (a), cardiac (c) and accessory (ac) lobes. (B) Severe lobar pneumonia with a firm grey-white lobulated tissue in the right apical lung lobe. (C) Irregular areas of grey-red consolidation in the right diaphragmatic lobe with (D) clear demarcation against normal lung tissue. (E) Generally enlarged mesenteric lymph nodes (n) with a humped surface. (F) Greatly enlarged tympanic bulla (b) of the left ear filled by pus.

The carcasses of the four muskoxen found dead and examined in 2012 (Table 1) were all decomposed and unsuitable for a thorough postmortem evaluation. Nevertheless, firm pneumonic lesions could be demonstrated in cranial parts of their lungs; three of them had fibrinous pleuritis and two had diarrhea. The lungs from the calf culled and autopsied in the field in 2012 and the lungs from the three calves culled and sampled in 2013 all showed a well-developed lobar pneumonia in the apical and cardiac lobes, being especially severe in the apical lobe on the right side (Fig. 3A–B). In the diaphragmatic lobes of the lungs, lesions occurred as irregular consolidations with clear demarcation against normal lung tissue (3C–D). Small amounts of mucopurulent exudate were seen on the cut surfaces and the pulmonary lymph nodes were generally swollen. In addition, the calf culled and autopsied in the field had swollen mesenteric lymph nodes (Fig. 3E) and the cut surfaces of both pulmonary and mesenteric lymph nodes showed the regular presence of grey-yellow foci of discoloration. The head submitted from one of the animals due to the presence of pus on one ear showed a left-sided otitis media with a greatly enlarged and thickened tympanic bulla filled with pus (Fig. 3F), as well as enlarged and abscessed retropharyngeal lymph nodes. From pus in the tympanic bulla *Pasteurella multocida* subsp. *multocida* was cultivated, whereas *M. ovipneumoniae* was identified by real-time PCR. Small numbers of lungworms (*Dictyocaulus* sp.) were detected in the airways of three of the animals examined.

**Figure 4:**
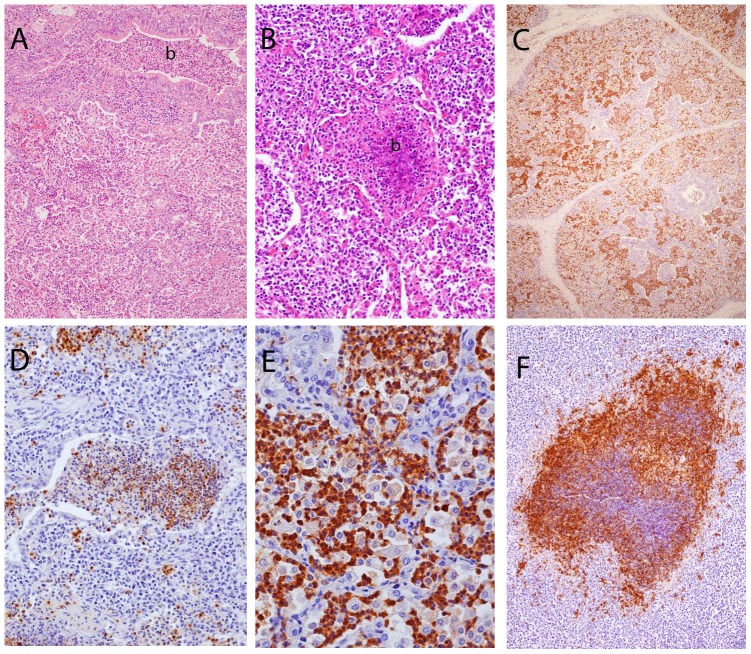
Microphotographs from sick muskoxen. (A) Inflamed lung tissue showing alveoli filled with macrophages and variable numbers of neutrophils, a bronchiole (b) lined by a slightly hyperplastic epithelium and filled with mixed cellular exudate, and moderate peribronchiolar accumulation of lymphoid cells. (B) Cell plug in a bronchiole (b) containing many necrotic neutrophils. (C) Severely consolidated lung tissue (from the apical lobe seen in 3B) with dense and widespread presence of brown-staining *Mycoplasma ovipneumoniae* antigens within alveoli and bronchioles. (D) *Mycoplasma ovipneumoniae* antigens in neutrophils and macrophages in a bronchiolar cell plug and elsewhere in the lung tissue. (E) Details of fig. 4C, showing intense staining of the cytoplasm of neutrophils and weak staining in macrophages. (F) Focal necrosis in a mesenteric lymph node with intensely positive IHC-staining in the periphery of the lesion. Note the presence of scattered stain-positive cells elsewhere in the lymphoid tissue. Haematoxylin and eosin stain (A, B) and immunoperoxidase method with haematoxylin counterstain (C, D, E, F).

Histopathological examination revealed a wide range of pulmonary lesions that could essentially be summarized as an acute to subacute mixed exudative and moderately proliferative bronchoalveolar pneumonia. Bronchioles and smaller bronchi showed a mild to moderate epithelial hyperplasia and slight peribronchial/peribronchiolar lymphoid hyperplasia; occasionally these lesions occurred as distinct lymphoid cuffs. Alveolar lesions included intra-alveolar exudation by macrophages and neutrophils, a mild type-2 pneumocyte hyperplasia (epithelialization), and leucocyte infiltration of alveolar septa (Fig. 4A). Fibrin was evident within the alveolar exudate in some of the lesions. Foci of aggregated necrotic cells (neutrophils) were occasionally seen and when occurring in bronchioles, they had the character of cell plugs (Fig. 4B). IHC examination of pneumonic lung sections revealed the specific staining of *M. ovipneumoniae* antigens in all animals examined, including the seven animals examined from the 2006 epidemic. The density of stain-positive materials varied considerably between sections, but severely infected areas (Fig. 4C) were observed in most animals. Staining was closely associated with lesions and was seen in the cytoplasm of neutrophils and macrophages (Fig. 4D), being especially strong in the neutrophils (Fig. 4E). Specific staining was not detected in control sections.

The pulmonary and all of the mesenteric lymph nodes histologically examined from the calf culled and autopsied in the field showed the presence of focal caseous necrosis that in some sites contained areas of calcification. All necrosis were strongly positive by IHC examination; staining being especially intense in the periphery of the lesions (Fig. 4F).

### Microbiology

**Figure 5:**
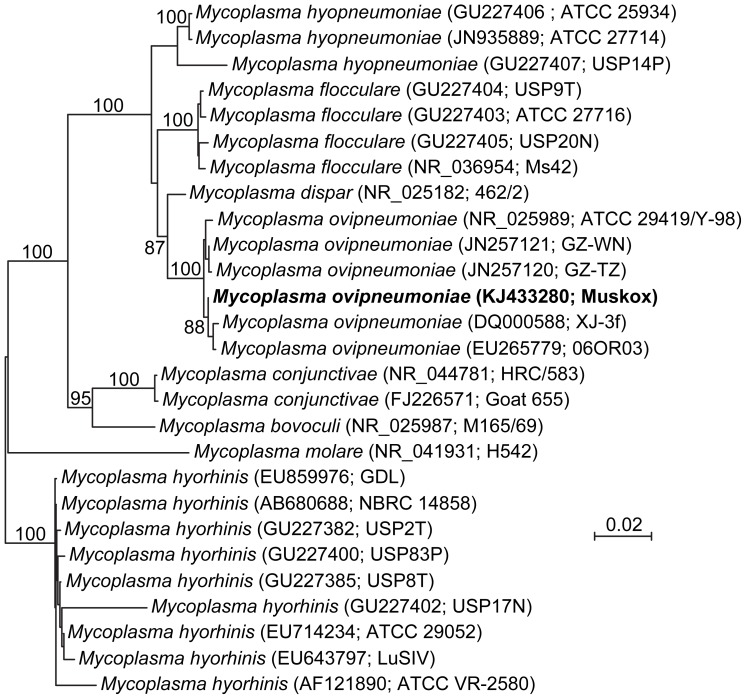
Phylogenetic tree. Neighbor-joining analysis of muskox mycoplasma isolates using Kimura 2-parameter distances and near full-length 16S rRNA sequences. Bootstrap values above 85% have been indicated (100 pseudo-replicates).

Pyrosequencing of pneumonic lung tissues from the six sick animals examined by this method (Table 2) yielded 635 826 reads and generated a total of 80 510 contigs. Sequence subtraction reduced the number of contigs to 18 986 (23.6%). Within this dataset, no candidate virus sequences could be identified. 1204 sequences matched the bacterial genome database when using megablast with word size 28. Results were ranked according to number of hits per bacterial genome and a list was compiled (Table S1). The top ranking species found in high concentrations in all of the six animals examined appeared to be a member of the *M. hyopneumoniae* group and a near complete 16S ribosomal RNA (rRNA) sequence could be constructed from the raw data and the contigs (GenBank accession number KJ433280). Phylogenetic analysis showed the sequence fell within a cluster of *M. ovipneumoniae* isolates (Fig. 5), most of which had been isolated from bighorn sheep (*Ovis canadensis*). The second ranked bacterium was *P. multocida multocida*, and this species was detected in four of the six animals examined.

The lung swabs from the 19 sick/dead animals examined for *M. ovipneumoniae* by real time PCR (Table 2) were all strongly positive (low Ct values), whereas only two of the 14 stray animals tested were positive. These two animals were killed in August/September 2013 and had relatively high Ct values (weak PCR signal).

Conventional bacteriology on lung swabs from the nine sick muskoxen examined (Table 2) revealed growth dominated by *P. multocida multocida* in four of the animals examined in 2012 and two of those examined in 2013. *Escherichia coli* and *Streptococcus bovis* were the dominating bacteria found in the remaining three animals. The four animals found culture positive for *P. multocida multocida* in 2012 were the same four that were positive for this bacterium by pyrosequencing.

*Mycoplasma ovipneumoniae* was successfully cultured from the lungs of five of the nine muskoxen examined.

### Serology

The results of the ELISA examination for *M. ovipneumoniae* are shown in table 3. Four (33%) of the ecthyma-sick calves culled in 2004 had antibodies whereas only one (4%) of the stray animals killed in the period prior to the 2006 epidemic tested positive. In the period between the two epidemics, 11 (19%) of the stray animals examined were antibody positive. All of the 10 pneumonic animals and nine (82%) of the stray animals culled during and after the 2012 epidemic were positive. The OD values were generally higher in sick muskoxen (mean: 0.79) as compared to seropositive stray animals (mean: 0.47) and ecthyma-sick calves (mean 0.49).

No seropositive animals were found among the 96 Greenlandic muskoxen examined, all showing low ODs, mean 0.10 (range 0.06–0.19).

### Sheep and reindeer

The results of the *Mycoplasma* culturing and serology in sheep are summarized in Table 4. *Mycoplasma ovipneumoniae* was isolated from 18 (45%) of the 40 animals examined, whereas *M. arginini* was cultivated from two. Twenty-eight of the sheep (70%) tested positive against *M. ovipneumoniae* by ELISA (mean OD 0.51); with a herd sero-prevalence varying between 50% and 100%. Interestingly, only 10 (56%) of the culture positive sheep were positive by serology.

**Table 4:** Sheep serology and *Mycoplasma* culturing.

Flock	Culture	Serology
No. 1	4/10	10/10
No. 2	4/10	6/10
No. 3	2/10	7/10
No. 4	8/10	5/10
In total	18/40	28/40

Number of sheep found positive/examined for *Mycoplasma ovipneumoniae* by culture and serology in four sheep flocks that had been on pasture in southern part of the muskox range during summer 2012.

None of the 35 reindeer examined showed positive ELISA values against *M. ovipneumoniae* (mean OD 0.08; range 0.04–0.23).

## Discussion

This study identified *M. ovipneumoniae* as a primary cause of the fatal pneumonia epidemic that occurred in the Norwegian muskox population in late summer 2012. Secondary invaders, in particular *P. multocida multocida*, detected in the lung tissue of a majority of the sick animals examined, presumably played an important role with regard to the course and fatal outcome of the pneumonia in many of the animals. Through retrospective IHC examination of archived lung tissues, we also identified *M. ovipneumoniae* as a likely primary cause of the pneumonia epidemic that occurred in this population in 2006 [4]. We also found serological evidence of *M. ovipneumoniae* infection in some of the calves culled during the ecthyma-outbreak that occurred in the muskox population in 2004 [3]. During this outbreak, the main focus was placed on grossly visible ecthyma lesions and cases of pneumonia may have been overlooked. The apparently low significance of *M. ovipneumoniae* infection in 2004 could be linked to a less virulent strain being present that year, causing subtle signs of pneumonia and little spread within the population. This assumption is supported by the fact that no serologically positive muskoxen were detected among the 23 stray animals shot between the autumn 2004 and start of the epidemic in 2006 (Table 3).

*Mycoplasma* spp. are regarded as highly host-specific bacteria that are not readily transmitted between animal species. There are however an increasing amount of reports of infection transmission between taxonomically closely related and even more distant host species [13], [14]. *Mycoplasma ovipneumoniae* is a sheep-adapted species commonly found in the airways of domestic sheep (*Ovis aries*) worldwide, including Norway [15] – [17]. In this natural host, it normally produces limited, chronic lung lesions and mild clinical signs [6], [16], [18]. However, following stressful events such as crowding and high summer temperatures, severe pneumonia may occur in lambs [16], [17], [19]. In some other ovine species, *M. ovipneumoniae* has been associated with severe fatal pneumonia epidemics following contact with domestic sheep. The first report was given for captive Dall's sheep (*Ovis dalli*) in Canada [20] and more recently, *M. ovipneumoniae* was identified as a primary cause of pneumonia epidemics in free-ranging bighorn sheep (*Ovis canadensis*); a devastating disease that has been recognized in bighorn populations in North America for centuries [21] – [23].

Mycoplasma infection has not previously been reported in the muskox and it is unlikely that this species is a natural host for *M. ovipneumoniae*. This was supported by the findings in this study, with no seropositive muskoxen detected among 96 animals examined from Kangerlussuaq in West Greenland, a population that, like the Norwegian population originates from animals translocated from native muskox areas in northeast Greenland [1], [2]. Our findings point to infection transmission into the Norwegian muskox population through contact with domestic sheep flocks that were present in the southern part of the muskox range during summer. The sheep in the four flocks examined after having been on this pasture during summer 2012, were commonly infected with *M. ovipneumoniae* (Table 4); 45% of the animals carrying this bacterium in their nose. Our findings indicate that sheep may carry this bacterium in their upper airways (nose) without seroconversion. Further molecular-biological studies are needed to compare the genetic relationship between the *M. ovipneumoniae* isolates from sheep and muskox. The reason why this agent is able to cause heavy disease outbreaks in the muskox could be due to different causes. One hypothesis could be that this high arctic species, ecologically and evolutionary adapted to regions without other ruminant species, except for reindeer (family Cervidae), has generally low resistance against infectious agents carried by domestic ruminants (sheep). Earlier studies have demonstrated that the muskox in Dovre is frequently infected with endoparasites picked up from co-grazing sheep and cattle [24]. Also the orf virus, causing the ecthyma outbreak in the muskox population in 2004 was most likely introduced from local sheep [3].

Mycoplasmas are minute wall-less bacteria possessing a very small genome and limited metabolic capacity, making them highly host dependent with a poor survival rate outside the host [14], [25]. Transmission of these agents therefore normally occurs through direct contact or via aerosols over short distances. Close interspecific contact is uncommon in the wild and grazing contact between sheep and muskox is not observed in Dovre, except for at the salt lick sites established for livestock (sheep, cattle) in the southern part of the muskox range. The muskox is recognized as a very eager user of these salt licks and have many times been observed chasing sheep away. These human-created contact sites can be considered suitable sites of infection transmission, both through aerosol exposure and indirect routes. Transmission via fresh secretions from the sheep's nose on the salt licks could be an important way of transmitting infection.

Presumably after a primary *M. ovipneumoniae* transmission from sheep to one or a small number of muskoxen, the infection apparently spread from muskox to muskox through close contact. The spread may have been exaggerated as a result of high population density and the ongoing rutting season (August/September), triggering male movements and increased contact between individuals and flocks. Arthropod vectors [26], [27] and weather conditions may also have contributed to spread. The 2006 outbreak coincided with a period of extraordinary hot and humid weather which was considered a major epidemiological factor [4]. In contrast during the 2012 outbreak, the weather was cool, although very humid [28]. The influence of these weather conditions is difficult to evaluate but may have influenced both the spread and severity of the *M. ovipneumoniae* infection and the role of secondary invaders. It has been observed [19] that a respiratory condition seen in lambs, principally caused by *M. ovipneumoniae* with *Actinobacillus pleuropneumoniae* as a common secondary invader, was associated with high temperatures during the summer.

Many *Mycoplasma* species show high degrees of genetic diversity which complicates the understanding of virulence, pathogenicity, immunology and the epidemiology of infection [14], [25]. For *M. ovipneumoniae* it has been shown that various strains often occur simultaneously within a sheep flock and even within individual animals [11], [29] – [31] whereas disease outbreaks are thought to be attributed to single strains [11]. Our findings point to separate infections introduced in 2006 and 2012 of a pathogenic strain from sheep that effectively spread within the muskox population. The introduced strain may also have changed in virulence through muskox passages during the outbreaks. The findings of calves and young adults being affected during the 2012 epidemic versus all age classes in 2006 indicated at least partial immunological protection among older animals following exposure in 2006. These assumptions are also supported by the fact that 19% of stray animals killed in the period between the two outbreaks were serologically positive (Table 3). In reality, this seroprevalence can be considered to be under-estimated due to the high ELISA cut off used in the present study (OD > 0.35), taking into consideration that none of the unexposed Greenlandic muskoxen showed OD-values above 0.19 as well as an expected reduction of titers over the six years period between the two outbreaks. In the stray animals killed and tested within a year after the 2012 outbreak, a seroprevalence of 82% was found. Little is known about the persistence of *M. ovipneumoniae* antibodies, except for a study in experimentally infected lambs, showing that good antibody titers are maintained for at least 16 weeks [32].

Whereas our findings clearly indicated that the 2006 strain was not maintained within the muskox population between the two outbreaks, the origin of infection of the few cases (three calves, one yearling) diagnosed in 2013 remains uncertain. These cases may have resulted from a new infection introduced from sheep during the summer of 2013. On the other hand, these cases might also reflect infection transmission from chronically infected adult animals (mother cows), established the previous year and maintained throughout the winter. Long duration of infection in chronically infected carrier animals is considered an important way of maintaining the infection in sheep flocks [11], [16], [17], and a mother to offspring transmission has been considered an explanation of lamb pneumonia seen in some bighorn sheep populations the next few years following a mycoplasma epidemic [21], [23].

The pathological evaluation of *M. ovipneumoniae* induced pneumonic lesions in muskoxen was complicated by concomitant infections by secondary invaders. However, based on the histopathological lesions seen in areas heavily infected with *M. ovipneumoniae*, as also confirmed by IHC, it could be concluded that this agent was a cause of extensive acute to subacute, exudative and in part purely suppurative bronchoalveolar inflammation. This is in contrast to this infection in its reservoir host domestic sheep, in which it normally produces small localized lesions characterized by chronic and proliferative inflammatory reactions [6], [16], [18].The high load of *M. ovipneumoniae*, as found in neutrophils by IHC in pneumonic muskox lungs indicated that these cells were highly involved in the clearing (phagocytosis) of this minute agent. Like bronchopneumonia in domestic animals [33], the lesions seen in muskoxen were especially pronounced in the cranial parts of the lungs. The finding that the most severe lesions were generally located in the right apical lobe could be attributed to this lobe in the muskox, like in domestic ruminants [34], being supported by the tracheal bronchus dividing from trachea markedly higher up as compared to the two principal bronchi, thus being closer to an ascending infection than the rest of the lungs.

An interesting finding in the muskox culled and autopsied in the field was generally swollen mesenteric lymph nodes which, like the pulmonary nodes contained foci of caseous necrosis that were invariably associated with large amount of *M. ovipneumoniae* antigens. Unfortunately, a thorough evaluation of the gastrointestinal tract of the remaining four whole muskox carcasses examined was not possible because of severe decomposition. Therefore, these findings should be evaluated with caution. Nevertheless, our findings indicate that *M. ovipneumoniae* is capable of establishing an intestinal infection in the muskox and it could be speculated that the diarrhea observed in some of the pneumonic animals was linked to this condition. Although various *Mycoplasma* spp. are generally associated with infections of the mucous membranes of specific organs in their natural hosts, with *M. ovipneumoniae* of sheep occurring in the airways, a switch in target organs has been reported when the organisms infect unnatural host species. For example the canine species *M. canis*, causing reproductive disease in dogs is associated with respiratory infection when transmitted to cattle [35] – [37]. Furthermore, some *Mycoplasma* species like *M. bovis* of cattle are associated with infection in multiple organ systems [38] and when transmitted to North American bison (*Bison bison*), it may cause severe disease outbreaks with systemic infection and multiple organ affections [39] – [41]. Interestingly, *M. bovis* infections are also associated with foci of caseous necrosis in various organs with a high load of the bacterium in the periphery of the lesions [38], [41], similarly to the necrotic foci seen in the lung tissue and pulmonary/mesenteric lymph nodes of muskox in this study.

Another observation made in this study was a severe suppurative otitis media in one animal linked to mixed infection by *M. ovipneumoniae* and *P. multocida multocida*. The head of this animal was submitted because of the presence of purulent exudate on one ear and no other animals underwent this examination. It is highly likely that otitis media (possibly also otitis interna) was involved in the disease pathogenesis of more animals in this study, including the animal observed with sideways drifting while moving. Otitis media has been associated with *M. ovipneumoniae* infection in bighorn sheep [21] and in cattle, regular outbreaks of *M. bovis* induced suppurative otitis media are reported [42]. A further spread from the middle ear to the inner ear and meninges of the brain may also occur [43].

In summary, this study identified *M. ovipneumoniae* as a plausible primary cause of the muskox pneumonia epidemics both in 2006 and 2012 and that domestic sheep were the likely source of infection. Molecular-biological studies will be carried out to compare the genetic relationship between the *M. ovipneumoniae* isolates from sheep and muskox. The outbreaks seemed to occur after two separate introductions of infection, the first leaving older animals immunologically protected against lethal infection during the second epidemic. Salt lick sites for sheep were considered a likely site of infection transmission, with subsequent spread from muskox to muskox. Care should be exercised when creating artificial contact points between animal species, especially between related species. Further research is needed to study if an introduced strain of *M. ovipneumoniae* from sheep can be maintained within the muskox population throughout the winter.

## Supporting Information

Table S1 The results of pyrosequencing of pneumonic lung tissues from six sick animals. Bacterial reference genome megablast hits^*^ using ‘non-muskox contigs’ and word size 28.
(DOCX)
