# Supplementary material for: Mycoplasma ovipneumoniae - A Primary Cause of Severe Pneumonia Epizootics in the Norwegian Muskox (Ovibos moschatus) Population
Source: PLoS One. 2014 Sep 8;9(9):e106116. doi: 10.1371/journal.pone.0106116 (PMC4157772; doi:10.1371/journal.pone.0106116)
Supplement: Table S1 — The results of pyrosequencing of pneumonic lung tissues from six sick animals. Bacterial reference genome megablast hits* using ‘non-muskox contigs’ and word size 28. (DOCX) [file pone.0106116.s001.docx]

Table S1. Results of pyrosequencing of pneumonic lung tissues.

| **Species** | **Acc. number** | **# of contigs*** |
| --- | --- | --- |
| Mycoplasma hyopneumoniae 168 | NC_017509.1 | 269 |
| Pasteurella multocida subsp. multocida | NC_017764.1 | 93 |
| Moraxella catarrhalis RH4 | NC_014147.1 | 63 |
| Escherichia coli 'clone D i14' | NC_017652.1 | 26 |
| Haemophilus parainfluenzae T3T1 | NC_015964.1 | 24 |
| Clostridium beijerinckii NCIMB 8052 | NC_009617.1 | 19 |
| Haemophilus parasuis SH0165 | NC_011852.1 | 17 |
| Salmonella enterica subsp. enterica serovar Weltevreden | NT_187124.1 | 17 |
| Clostridium botulinum B Eklund 17B(NRP) | NC_018648.1 | 16 |
| Pasteurella multocida 36950 | NC_016808.1 | 15 |
| Escherichia coli O104:H4 2009EL-2071 | NC_018661.1 | 15 |
| Thermococcus sp. 4557 | NC_015865.1 | 14 |
| Escherichia coli Xuzhou21 | NC_017906.1 | 11 |
| Mycoplasma conjunctivae HRC/581 | NC_012806.1 | 10 |
| Prevotella ruminicola 23 | NC_014033.1 | 10 |
| Chitinophaga pinensis DSM 2588 | NC_013132.1 | 9 |
| Clostridium perfringens SM101 | NC_008262.1 | 8 |
| Clostridium botulinum E3 Alaska E43 | NC_010723.1 | 8 |
| Streptomyces flavogriseus ATCC 33331 | NC_016114.1 | 8 |
| Actinobacillus suis H91-0380 | NC_018690.1 | 8 |
| Propionibacterium acnes C1 | NC_018707.1 | 8 |
| Psychrobacter sp. PRwf-1 | NC_009524.1 | 7 |
| Yersinia enterocolitica subsp. palearctica 105 | NC_015224.1 | 5 |
| Escherichia coli KO11FL | NC_017660.1 | 5 |
| Acinetobacter baumannii TYTH-1 | NC_018706.1 | 5 |
| Pasteurella multocida subsp. multocida str | NC_017027.1 | 4 |
| Mycoplasma hyorhinis SK76 | NC_019552.1 | 4 |
| Haemophilus influenzae PittGG | NC_009567.1 | 3 |
| Haemophilus influenzae F3047 | NC_014922.1 | 3 |
| Acinetobacter calcoaceticus PHEA-2 | NC_016603.1 | 3 |
| Bradyrhizobium japonicum USDA 6 | NC_017249.1 | 3 |
| Haemophilus influenzae R2846 | NC_017452.1 | 3 |
| Escherichia coli NA114 | NC_017644.1 | 3 |
| Escherichia coli O7:K1 CE10 | NC_017646.1 | 3 |
| Escherichia coli O55:H7 RM12579 | NC_017656.1 | 3 |
| Secondary endosymbiont of Heteropsylla cubana | NC_018420.1 | 3 |
| Pasteurella multocida subsp. Multocida | NC_002663.1 | 2 |
| Rhodopseudomonas palustris HaA2 | NC_007778.1 | 2 |
| Prevotella melaninogenica ATCC 25845 | NC_014371.1 | 2 |
| Clostridium cellulovorans 743B | NC_014393.1 | 2 |
| Bacteroides helcogenes P 36-108 | NC_014933.1 | 2 |
| Prevotella denticola F0289 | NC_015311.1 | 2 |
| Porphyromonas asaccharolytica DSM 20707 | NC_015501.1 | 2 |
| Bacillus coagulans 2-6 | NC_015634.1 | 2 |
| Capnocytophaga canimorsus Cc5 | NC_015846.1 | 2 |
| Neisseria meningitidis WUE 2594 | NC_017512.1 | 2 |
| Escherichia coli O83:H1 NRG 857C | NC_017634.1 | 2 |
| Prevotella intermedia 17 | NC_017861.1 | 2 |
| Klebsiella oxytoca E718 | NC_018106.1 | 2 |
| Mycoplasma genitalium M2321 | NC_018495.1 | 2 |

*Only genomes with two or more matching contigs have been listed.
